# Supplementary material for: Emergent anisotropy in the Fulde–Ferrell–Larkin–Ovchinnikov state
Source: Nat Commun. 2022 Oct 3;13:5590. doi: 10.1038/s41467-022-33354-1 (PMC9530125; doi:10.1038/s41467-022-33354-1)
Supplement: Supplementary file 1 — Supplementary Information [file 41467_2022_33354_MOESM1_ESM.pdf]

Supplementary Materials for
Emergent anisotropy in the Fulde–Ferrell–Larkin–Ovchinnikov state

Shusaku Imajo*, Toshihiro Nomura, Yoshimitsu Kohama, and Koichi Kindo
Institute for Solid State Physics, University of Tokyo, Kashiwa 277-8581, Japan

Table of Contents

§1 Details of our multidirectional ultrasound measurements
§2 Pulsed magnetic field
§3 Acoustic de Haas oscillations
§4 Hysteresis at H_{FFLO}
§5 Magnetic field dependence of $\Delta v/v$ in parallel fields at various temperatures
§6 Out-of-plane electrical resistance
Supplementary Figure 1-6
Supplementary References

1. Details of our multidirectional ultrasound measurements

In this study, we implemented multidirectional ultrasound measurements using four $LiNbO_3$ piezoelectric transducers. Supplementary Figure 1a shows a photo of the sample setup for the measurements. The sample shape is indicated by the red dotted line, and the transducers are highlighted in blue. The gold wires in the picture are the electrical wiring to the transducers to apply and detect sound waves. Rotating magnetic fields are applied in the *a**-*c* plane. As shown in Supplementary Fig. 1b, the crystal used in this study was sliced and formed into a square shape to attach the transducers with epoxy resin because typical crystals of κ-(BEDT-TTF)$_2$Cu(NCS)$_2$ were obtained in a hexagonal shape. Longitudinal sound waves were generated from one of the transducers and detected from another. The sound velocity v is approximately 2.4 km/s at 10 K and has no significant in-plane anisotropy. Supplementary Figure 1c shows the temperature dependence of $\Delta v/v$ for the superconducting state (0 T) and the normal state (5 T, applied perpendicular to the conducting plane) of κ-(BEDT-TTF)$_2$Cu(NCS)$_2$. The data are well consistent with the reported data (1). Notably, measuring the temperature dependence with the above pulsed-field ultrasound measurement setup was difficult due to the temperature control, and therefore, only the temperature dependence was measured using another probe and another crystal in a static field.

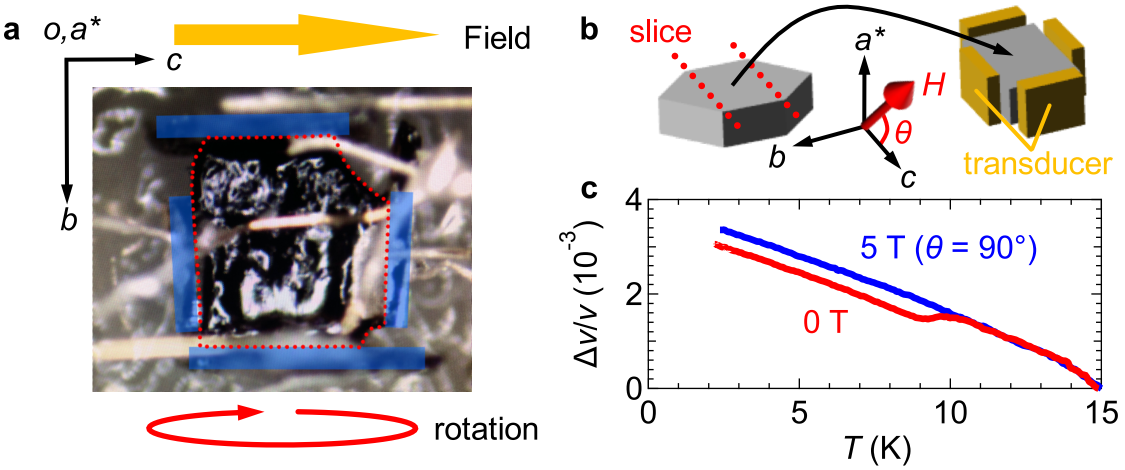

Supplementary Figure 1: a Photo of the sample setup with piezoelectric transducers (blue). The red dotted curve emphasizes the outline of the sample. Rotating magnetic fields are applied in the *a**-*c* plane. **b** Schematic illustration of the treatment for attaching the transducers to the crystal. **c** Temperature dependence of $\Delta v/v$ at 0 T and 5 T when $\boldsymbol{u} \| b$ and $\boldsymbol{H} \| a^*$.

2. Pulsed magnetic field

All the data in the main text are obtained by using pulsed magnetic fields, which were generated by an in-house pulse magnet. In Supplementary Fig. 2, field profiles of typical pulsed magnetic fields used in this study are shown. The duration of the pulsed fields was about 38 ms. Using a 0.9 MJ capacitor bank (18 mF), the 33.8 T and 51.9 T pulsed fields were generated with charging voltages of 5 kV and 8 kV, respectively.

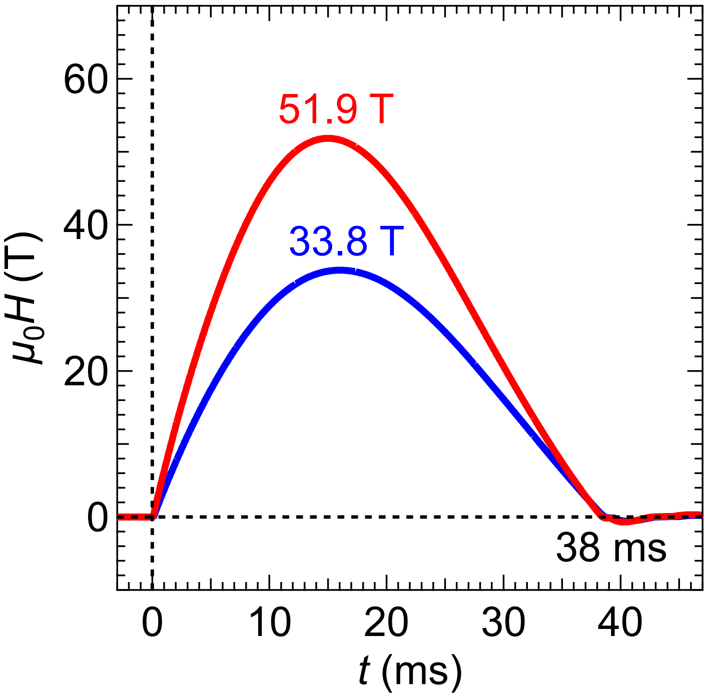

Supplementary Figure 2: Field profiles of 33.8 T and 51.9 T pulsed-field shots with the duration of ~38 ms.

3. Acoustic de Haas oscillations

Supplementary Figure 3a shows the oscillatory part of $\Delta\alpha$, $\Delta\alpha_{osc}$, originating from the acoustic de Haas oscillation at 1.6 K. The black curve is a fit to the Lifshitz-Kosevich formula with the frequencies of the α orbit F_α=610 T and the β−α orbit $F_{\beta-\alpha}$=3300 T. The Fourier spectrum of $\Delta\alpha_{osc}$ is shown in Supplementary Fig. 3b. This spectrum clearly indicates the absence of the β orbit component with F_β=3900 T and the presence of the β−α orbit, known as the forbidden orbit, component in the acoustic de Haas signal. The absence of the β orbit component is reasonable since the measurement temperature is relatively high (T>1.6 K) to detect the heavy-mass β orbit (m_β~6m_e). Supplementary Figure 3c shows the mass plot in ln(A/T) vs. T format, where A represents the Fourier amplitude estimated in the range from 30 T to 50 T. Both orbits show similar temperature dependence, and an effective mass of m^*~3.0m_e is obtained for both. For the α orbit, the result shows good agreement with the reported data, m_α~3.2m_e (2,3). However, the value of $m_{\beta-\alpha}$ strongly depends on the measurement technique (2,3). The origin and features of the β−α orbit are still open questions.

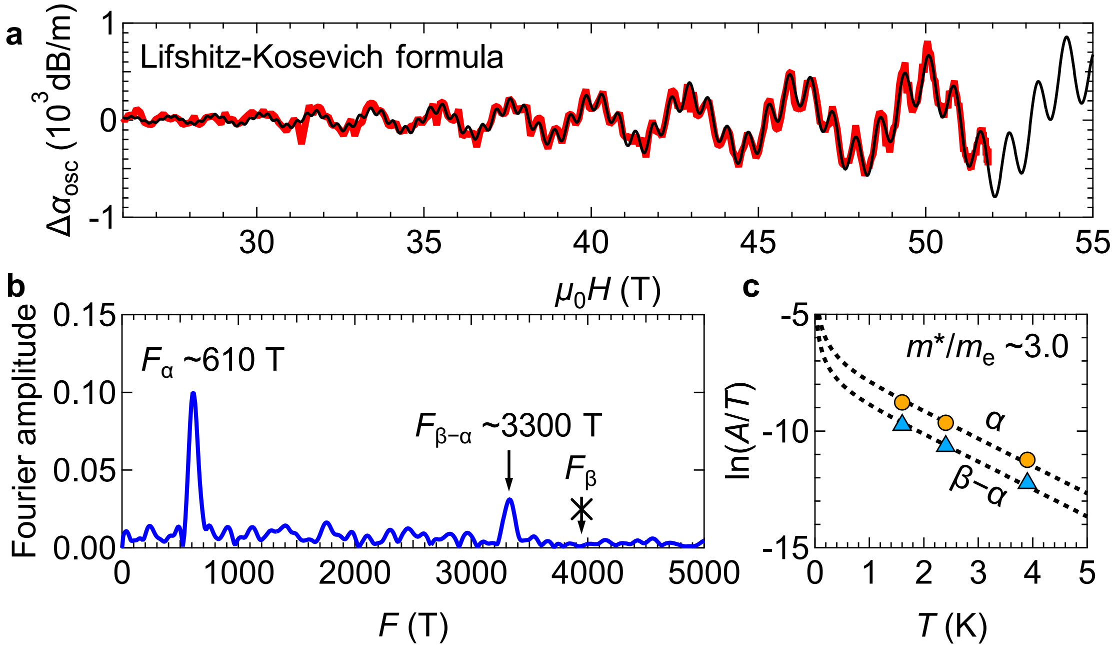

Supplementary Figure 3: a Acoustic de Haas oscillation observed in $\Delta\alpha$ at 1.6K. The black curve represents a fit to the two-component Lifshitz-Kosevich formula of the α orbit and the β−α orbit. **b** Fourier spectrum of the acoustic de Haas shown in **a**. The peaks are observed at the frequencies F_α=610 T and $F_{\beta-\alpha}$=3300 T. The reported frequency for the β orbit $F_{\beta-\alpha}$=3900 T (2) was not detected in the present measurement due to the relatively high temperature. **c** Mass plot of the detected orbits. Both are well reproduced with effective mass m^*~3.0m_e, which is consistent with the reported data m_α~3.2m_e (2,3).

5. Hysteresis at H_{FFLO}

The order parameter of the FFLO state includes the term of the **q** vector, cos(**qr**), which discontinuously jumps from zero to a finite value at H_{FFLO}. Therefore, the phase transition between the ordinary superconducting state and the FFLO state is a first-order one. Indeed, earlier studies demonstrate (4,5) that the transition at H_{FFLO} in κ-(BEDT-TTF)$_2$Cu(NCS)$_2$ is a first-order transition showing a hysteresis. The hysteresis is clear only at very low temperatures, and becomes less clear above 1.5 K (4,5). Supplementary Figure 4 shows the ultrasound data at θ=0° in up- and down-sweep fields at 1.6 K, which is the lowest temperature in our measurements. In these datasets, no clear hysteresis is observed, which is consistent with the previously reported data above 1.5 K.

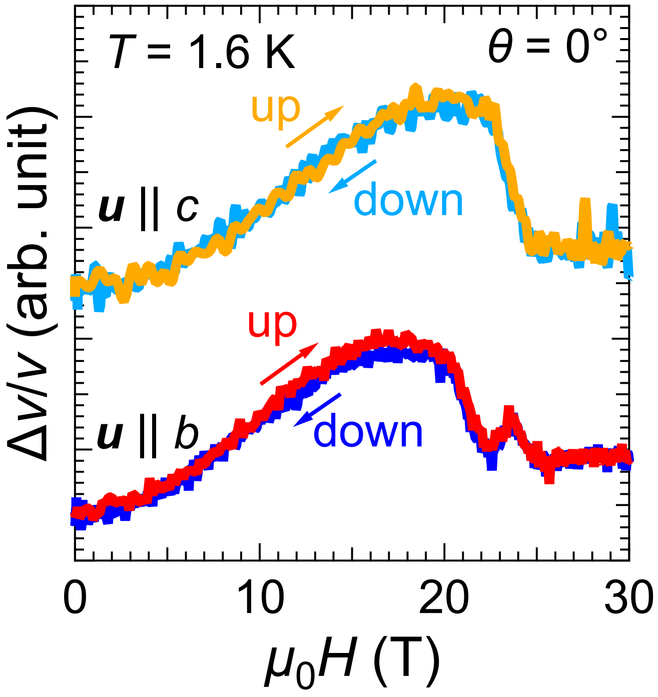

Fig. S4 Field dependence of $\Delta v/v$ (θ=0°) at the lowest temperature, 1.6 K, in up- (red and orange) and down-sweeps (blue and light blue).

5. Magnetic field dependence of $\Delta v/v$ in parallel fields at various temperatures

The data points $H_{c2}(\theta=0°)$ shown in Fig. 3 are obtained from the field dependence of $\Delta v/v$ at various temperatures shown in Supplementary Fig. 5. Above 4 K, an additional anomaly is observed at ~5 T. Since elastic properties are strongly influenced by vortices, this anomaly should be attributable to transitions of the vortices. The Josephson vortex lattices in 2D layered superconductors exhibit several transitions related to their depinning and melting, as reported (6). The drastic enhancement of $\Delta v/v$ below 5 T is most likely due to pinning of the flux lines since this anomaly disappears at low temperatures. This is because the interlayer coherence length in 2D superconductors becomes smaller than the interlayer spacing at lower temperatures, and the pinning effect on the Josephson vortices in the insulating layers is strongly suppressed due to interlayer decoupling. For depinned Josephson vortices, the flux-line lattice flows and can be easily deformed in the insulating plane; and therefore, the in-plane anisotropy should be reduced. This suppression of the in-plane anisotropy should correspond to the small sound wave direction dependence below H_{FFLO}, as shown in Fig. 4a.

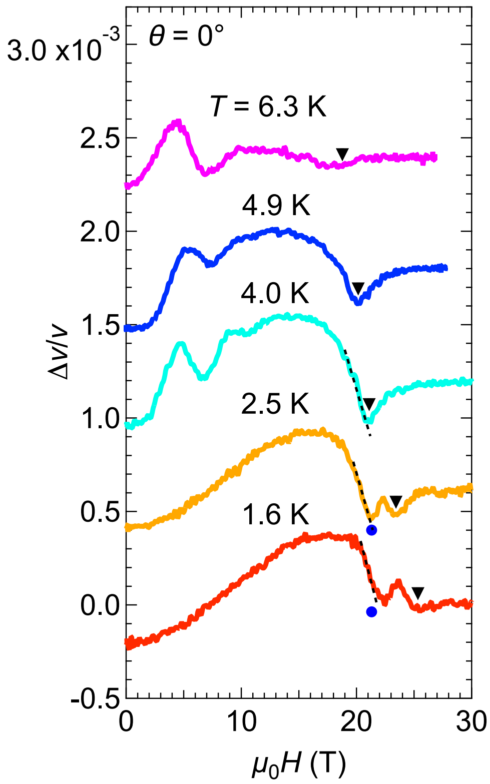

Supplementary Figure 5: In-plane magnetic field dependence of $\Delta v/v$ at various temperatures. The dashed lines are guides to make the transition at H_{FFLO} (blue circle) clearer.

6. Out-of-plane electrical resistance

During the ultrasound measurements, we additionally measured the out-of-plane electrical resistance of another crystal at the same time. The field directions were the same as those shown in Supplementary Fig. 1 and electric currents were applied parallel to the *a**-axis. Supplementary Figure 6 shows the magnetic field dependence of the resistance at 1.6 K. Since these were just supplementary measurements obtained while performing the main ultrasound measurements, there are no data at $|\theta|$=0°. Nevertheless, the data at $\theta = -0.7°$ and $-0.5°$ show the resistivity data of the FFLO state appearing above H_{FFLO}=21 T. H_{c2} is determined by the flection points shown by the pink triangles. As indicated by the arrows, these data show some kinks in the FFLO state. Since the finite resistance originates from the vortex dynamics, these kinks should be related to vortex pinning. Similar kink structures of the out-of-plane resistance are reported in other FFLO candidates (6,7) and are regarded as the commensurability effect of pinning on the FFLO spatial modulation (6-8). This effect occurs at certain fields where the FFLO wavelength $2\pi/q$ is commensurate with the Josephson vortex lattice constant d_{JV}, which leads to smaller resistances due to the relatively strong pinning at the nodes. This effect also indicates that the **q** vector is parallel to the *b*-axis because the spatial modulation needs to trap the flux lines, which is consistent with the results obtained by our ultrasound measurements.

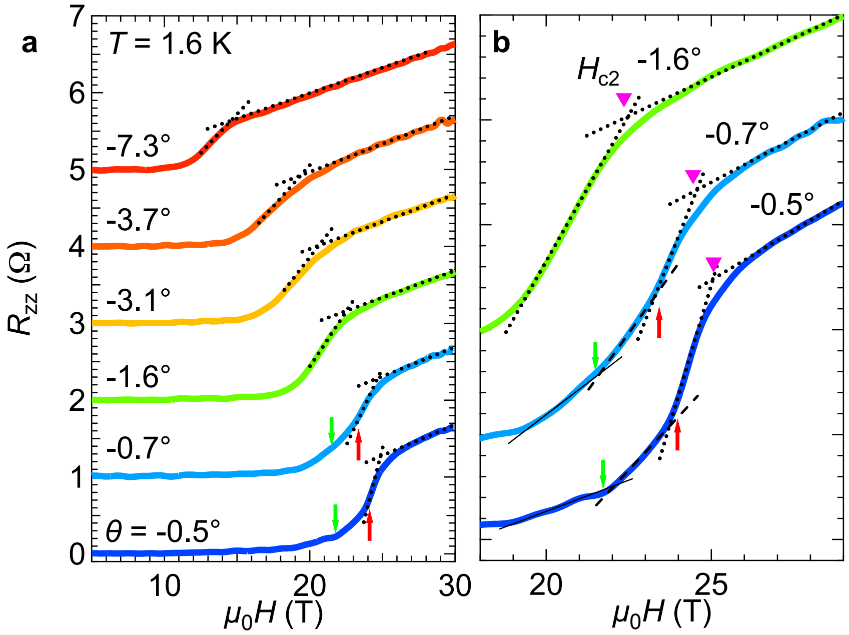

Supplementary Figure S6: a,b Out-of-plane magnetoresistance at 1.6 K at various angles. An enlarged plot of the datasets for θ=−1.6°, −0.7°, and −0.5° is shown in **b**. The pink triangle shows H_{c2} at each angle. The arrows point to kinks in the FFLO state.

Supplementary References

[1] Yoshizawa, M., Nakamura, Y., Sasaki, T., & Toyota, N. Sound velocity change at superconducting transition in κ-(BEDT-TTF)$_2$Cu(NCS)$_2$. *Solid State Commun.* **89**, 701 (1994).

[2] Uji, S., Chaparala, M., Hill, S., Sandhu, P. S., Quails, J., Seger, L., & Brooks, J. S. Effective mass and combination frequencies of de Haas-van Alphen oscillations in κ-(BEDT-TTF)$_2$Cu(NCS)$_2$. *Synth. Met.* **85**, 1573, (1997).

[3] Hill, S., Uji, S., Sandhu, P. S., Brooks, J. S. & Seger, L. A comparison of the high field quantum oscillations observed by electrodynamic and d.c. transport techniques in the organic superconductor κ-(BEDT-TTF)$_2$Cu(NCS)$_2$. *Synth. Met.* **86**, 1955 (1997).

[4] Agosta, C. C., Fortune, N. A., Hannahs, S. T., Gu, S., Liang, L., Park, J.-H., & Schleuter, J. A. Calorimetric Measurements of Magnetic-Field-Induced Inhomogeneous Superconductivity Above the Paramagnetic Limit. *Phys. Rev. Lett.* **118**, 267001 (2017).

[5] Bergk, B., Demuer, A., Sheikin, I., Wang, Y., Wosnitza, J., Nakazawa, Y., & Lortz, R., Magnetic torque evidence for the Fulde-Ferrell-Larkin-Ovchinnikov state in the layered organic superconductor κ-(BEDT-TTF)$_2$Cu(NCS)$_2$. *Phys. Rev. B* **83**, 064506 (2011).

[6] Sugiura, S., Terashima, T., Uji, S., Yasuzuka, S., & Schlueter, J. A. Josephson vortex dynamics and Fulde-Ferrell-Larkin-Ovchinnikov superconductivity in the layered organic superconductor β''-(BEDT−TTF)$_2$SF$_5$CH$_2$CF$_2$SO$_3$. *Phys. Rev. B* **100**, 014515 (2019).

[7] Uji, S., Terashima, T., Nishimura, M., Takahide, Y., Konoike, T., Enomoto, K., Cui, H., Kobayashi, H., Kobayashi, A., Tanaka, H., Tokumoto, M., Choi, E. S., Tokumoto, T., Graf, D., & Brooks, J. S. Vortex Dynamics and the Fulde-Ferrell-Larkin-Ovchinnikov State in a Magnetic-Field-Induced Organic Superconductor. *Phys. Rev. Lett.* **97**, 157001 (2006).

[8] Bulaevskii, L., Buzdin, A., & Maley, M. Intrinsic Pinning of Vortices as a Direct Probe of the Nonuniform Larkin-Ovchinnikov-Fulde-Ferrell State in Layered Superconductors. *Phys. Rev. Lett.* **90**, 067003 (2003).
